# Comparison of linear mixed models for genetic feather score analysis in laying hens kept in recurrent testing facilities

**DOI:** 10.1016/j.psj.2025.104833

**Published:** 2025-01-20

**Authors:** T. Osorio-Gallardo, P. Bijma

**Affiliations:** Animal Breeding and Genomics Centre, Wageningen University, 6709PG Wageningen, the Netherlands

**Keywords:** Feather score, Recurrent testing, Validation, Model comparison, Genetic analysis

## Abstract

Feather pecking, related feather damage, and mortality are crucial welfare and efficiency traits in laying hens. When individuals are kept in sib groups, genetic analysis of feather scores captures the performer and receiver components of feather damage due to pecking. Genetic parameters and breeding values estimated from such data with an ordinary linear mixed model refer to total breeding values. Thus, breeding against feather pecking-related mortality is possible by selecting breeding values for feather score when these are estimated from sib-groups. “Feather score” is part of the selection indexes of some breeding companies. However, there is no public information on the extensive evaluation and validation of the models used. Moreover, survival and feather score are genetically correlated, potentially biasing feather score-breeding value and genetic parameters estimation. This study compared and validated six models for genetic analysis of feather scores in the back and neck regions of laying hens at 45 and 70 weeks of age. We tested univariate models of feather score along with bivariate models for feather score and survival, and both sire and animal models, using individual or cage-level records. We compared the performance of the models based on the accuracy and dispersion of estimated breeding values. Additionally, genetic variances for all traits were estimated and compared. The individual-level univariate animal model showed the poorest performance for both accuracy and dispersion. Apart from the previous, no clear superiority regarding accuracy was observed between animal and sire models, nor between univariate and bivariate models. Breeding values estimated from cage-level observations tended to show less over-dispersion, and the estimates from the univariate cage-level sire model showed no significant over-dispersion for all traits. Since the univariate cage-level sire model was the simplest, as accurate as any of the other models, and showed no over-dispersion, it was considered the best model for feather score analysis with recurrent testing data.

## Introduction

Feather pecking (**FP**) is a major welfare issue in commercial laying hens and can result in the loss/damage of feathers, injuries and damage of the skin, and even in the death of the victim (Savory, 1995). Since the prohibition of individual cages in the European Union in 2012, FP has increased, and diverse control strategies like: dimming the light or using red light (Shi et al., 2019), the placement of an “isolation chamber” to protect severely pecked animals (Bist et al., 2023), or the supplementation of tryptophan in feed (van Hieu et al., 2022) have been tested and investigated.

Genetic selection against FP has been studied for several years, including selection for beak shape to mitigate FP (Icken et al., 2017), genomic studies to identify DNA regions related to FP (Iffland et al., 2020), and the possibility of using sensor technology for FP phenotyping (Ellen et al., 2019).

However, to study FP, the event of FP must be observed, which is very labor intensive. For this reason, several studies used feather cover score or feather damage score (from here on referred to as “feather score” (**FS**)) as an indirect phenotype for FP because the FS of an individual is strongly related to receiving severe FP (Bilcik and Keeling, 1999). FS is typically scored as a categorical variable reflecting the feather damage on the neck, back, rump and/or belly of the individual because feather damage in these areas is most likely due to severe FP (Rodenburg et al., 2010; Brinker et al., 2014; Decina et al., 2019).

When FS is recorded on birds kept in groups of unrelated individuals, genetic analysis of FS records with an ordinary animal or sire model captures only the victim components of FP. In other words, it only captures the genetic effects on the propensity of individuals to receive FP and gives no information on the genetic propensity of individuals to perform pecking (the “social genetic effect”) (Muir, 2005).

Sun et al. (2014) analyzed feather scores obtained from mixed groups of half and full sisters of White Leghorns, and estimated variance components using both a traditional animal model and a direct-associative model. The total heritable variance of FS estimated with the direct-associative model was twice as large as the additive genetic variance estimated with the traditional animal model. Similarly, in Brinker et al. (2014), the estimated total heritable variance of FS was more than five times larger than its traditional heritability. These results suggest that most of the heritable variation in FS originates from social genetic effects and show the need for breeding methods that capture both the direct and social genetic effect underlying FS.

In layer breeding, the so-called recurrent testing (**RT**) is a common practice to evaluate the performance of sires of laying hens. For these tests, daughters of the same sire are grouped together in sib groups and information on traits like egg production and mortality are recorded. When individuals are kept in sib groups, the genetic analysis of individual FS records captures both the receiver and the performer components of FP. For this reason, the genetic parameters and estimated breeding values (**EBV**) from an ordinary linear mixed model applied to RT data refer to the total breeding value (Bijma et al., 2007; Ellen et al., 2007). Hence, to breed against feather pecking, it is not necessary to identify the peckers when groups consist of sibs.

FS is part of the selection indexes of some breeding companies (Braak, 2019a, 2019b). However, to the author's knowledge, information on evaluation and validation of the models used for variance components and breeding value estimation is not available in the scientific literature. Moreover, survival and FS are genetically correlated (Brinker et al., 2014). On the one hand, this means that mortality due to FP can be reduced by selection for better FS (Muir and Craig, 1998; Ellen and Bijma, 2019). On the other hand, FS-related mortality may lead to bias in estimated genetic parameters and breeding values for FS because of survival-related selection in the data. In other words, particularly when FS is recorded later in life, part of the population will have no records because they died before FS recording. Hence, as FS records come from a population preselected for a trait (survival) correlated to FS, the genetic estimates can be biased when the genetic effects for FS are estimated univariately (Robertson, 1977).

For this reason, in the present study, we compared and validated six different models for genetic analysis of FS using RT data and observed their predictive performance. We tested univariate models of FS as well as bivariate models for FS and survival, and both sire and animal models, either with individual records or cage-level records. We described the differences between the performance of these models as a way to assess their suitability for genetic improvements of FS.

## Material and methods

### Data sets

Hendrix Genetics® (**HG**) provided data on FS and survival of individuals descending from a single sire line that were kept in their RT facilities. The name of the sire line was not disclosed. This section gives a description of the raw data, and the edits used to obtain the final data for the analysis. Table 1 shows descriptive statistics of the final data.

**Table 1:** Data set description.

Descriptive statistics of working data	
Number of individuals	111,761
Number of cages	12,650
Number of sires	2,104
Number of farms	20
Number of dam lines	8
Number of individuals per cage	3 to 22
Years	2014 to 2021
Mean survival percentage at 45 weeks	98 %
Mean survival percentage at 70 weeks	94 %
Mean FS for BACK45	11.32
Mean FS for NECK45	12.42
Mean FS for BACK70	13.98
Mean FS for NECK70	14.78

BACK45: feather score of the back at 45 weeks of age; BACK70: feather score of the back at 70 weeks of age; NECK45: feather score of the neck at 45 weeks of age; NECK70: feather score of the neck at 70 weeks of age.

***Raw Data*.** The received FS data included the number of individuals in each RT cage that had a specific FS for the body areas: neck and back at 45 weeks and 70 weeks of age. This resulted in four traits: NECK45, BACK45, NECK70 and BACK70. FS was recorded by HG as a three-level categorical variable, where 10 was perfect feather coverage, 15 meant the presence of damaged feathers, and 20 meant the presence of bald patches (M. Faure, personal communication, February 10, 2023). Hence, FS records were not linked to the individual ID, but for each of the three scores (10, 15 or 20), the number of individuals for each score were recorded for each cage. This resulted in three records for each of the four traits for each cage. For example, for BACK45 in a cage of ten individuals, six may have been scored with 10, three with 15, and one with 20. Therefore, for that cage, the data would have reported: BACK45, score 10 – 6 individuals, score 15 – 3 individuals, and score 20 - 1 individual.

The survival data included the original number of individuals in each cage, their date of housing, date of death, removal, or end date of the batch, and the individual ID. All individuals in a cage were crossbreds; they shared the same sire and had an unknown dam from a single dam line. Each sire had been mated to around 18 to 20 dams. Thus most cage mates were half-sib, but some may have been full-sibs. However, as there was no information on the identity of the dam, the proportion of half-sib / full-sib in each cage was unknown.

The data also provided the name of each RT facility (from here on “farm”), the row number, the cage number, the cross-code, the sire ID, the genotype for a fast feathering gene, and the identification number of each recurrent test (RT number). The RT number was a combination of numbers given to identify the batch, farm, and row number of each of the recurrent tests. All of the aforementioned information was identical for all members of the same cage.

***Working Data Sets.*** RStudio “2023.06.1 + 524″ (Posit team, 2023) software for Windows was used for all editing and descriptive analyses.

Two working data sets were created. The first had cage-level records, each reflecting the cage average for one of the four FS traits. Survival for each cage was calculated as the number of individuals alive at the moment of feather scoring divided by the original number of individuals. Also, the ID of a single random member of the cage was included to be used in the cage-level univariate animal model later on (see **CUAM** below). Information about the farm, RT number, cage number, sire ID, feathering gene genotype, cross code, housing date, and removal date was also kept.

The second data set was designed so that each individual of every cage, alive at the moment of scoring, had its “own” FS record. Individuals dead at that moment of scoring were given “NA” (not available). This data set was created from the number of individuals with each of the three scores that were available in the raw data. For example, for a cage that housed nine birds, the raw data may have indicated four individuals with a score of 10, three with a score of 15, one with a score of 20, and one individual who died before scoring. As the non-surviving bird had no FS record, in the data set the FS record for this individual was coded as “NA”. Next, from the eight surviving birds, four random individuals were given a score of 10, three were given a score of 15, and the remaining one was given a score of 20. While the scores allocated may differ from the actual scores of the individuals, the resulting data is fully equivalent for genetic analysis because all cage members had the same pedigree (and no offspring or genotypes). Survival for each individual was coded as “2″ when alive and “1″ when dead at the moment of feather scoring. All individual IDs were kept. The rest of the information was equivalent to dataset one and identical for all cage members.

### Models

Six different linear mixed models were used to estimate genetic parameters and breeding values for each of the FS traits. To establish which fixed effects to include in the model, a linear model was fitted in R. Fixed effects were identical across models, and included the following: First, the RT number to correct for infrastructural and time effects (i.e., the difference in lightning and year/season). Second, an interaction of farm by housing date by cross. Finally, the genotype for a feathering speed gene. The models were then extended with random effects in ASREML 4.2.1 for Linux (Gilmour et al., 2015). To account for non-genetic effects shared by members of the same cage, a random cage effect was included in individual-level models.

To test whether the genetic effects of FS were affected by pre-selection on survival, both univariate models of FS and bivariate models of FS and survival were fitted. Given that all cage members had the same sire and an unknown dam, it was more straightforward to fit sire models for the estimation of variance components. However, as animal models are commonly used in animal breeding, we also evaluated animal models.

***Animal models.*** The following three animal models were fitted:

1. Individual level bivariate animal model (**IBAM**)
  $$\left[\begin{array}{c} y_{1}\\ y_{2} \end{array}\right]=\;\left[\begin{array}{cc} X_{1} & 0\\ 0 & X_{2} \end{array}\right]\left[\begin{array}{c} b_{1}\\ b_{2} \end{array}\right]+\left[\begin{array}{cc} Z_{1} & 0\\ 0 & Z_{2} \end{array}\right]\left[\begin{array}{c} a_{1}\\ a_{2} \end{array}\right]+\left[\begin{array}{cc} V_{1} & 0\\ 0 & V_{2} \end{array}\right]\left[\begin{array}{c} c_{1}\\ c_{2} \end{array}\right]+\left[\begin{array}{c} e_{1}\\ e_{2} \end{array}\right]$$
  Where subscript 1 refers to an FS trait and subscript 2 to survival; **y** is a vector of individual records, **X** is an incidence matrix connecting records to fixed effects; **b** is the vector of fixed effects; **Z** is an incidence matrix connecting each individual's phenotype to its own breeding value; **a** is the vector of breeding values; **V** is an incidence matrix connecting records to random cage effects; **c** is a vector of independent random cage effects; and **e** is a vector of residuals.
  The distribution of the genetic effects was:
  $$\left[\begin{array}{c} a_{1}\\ a_{2} \end{array}\right]=N\left(\left(\begin{array}{c} 0\\ 0 \end{array}\right),\;\left[\begin{array}{cc} \sigma_{A_{1}}^{2} & \sigma_{A_{1}A_{2}}\\ \sigma_{A_{1}A_{2}} & \sigma_{A_{2}}^{2} \end{array}\right]⊗A\right)$$
  Where $\sigma_{A_{1}}^{2}$ was the genetic variance of the FS trait, $\sigma_{A_{2}}^{2}$ was the genetic variance of survival, $\sigma_{A_{1}A_{2}}$ was the genetic covariance between the traits, ⊗ denoted the Kronecker product of matrices, and **A** was the matrix of additive genetic relationships between individuals, based on 21 generations of pedigree information.
  The distribution of the cage effects was:
  $$\left[\begin{array}{c} c_{1}\\ c_{2} \end{array}\right]=N\left(\left(\begin{array}{c} 0\\ 0 \end{array}\right),\;\left[\begin{array}{cc} \sigma_{c_{1}}^{2} & \sigma_{c_{1}c_{2}}\\ \sigma_{c_{1}c_{2}} & \sigma_{c_{2}}^{2} \end{array}\right]⊗I\right)$$
  The distribution of residuals was analogous to that of the cage effects.
2. Individual-level univariate animal model (**IUAM**)
  **y**_1_ = **Xb** + **Za** + **Vc** + ***e***
  The model components were similar to those for FS in IBAM. The distribution of the random effects was $a∼\;N(0,\;A\sigma_{A}^{2}),$ $c∼\;N(0,\;I\sigma_{c}^{2})$, and $e∼\;N(0,\;I\sigma_{e}^{2})$.
3. Cage level univariate animal model (**CUAM**)
  **y**_1_ = **Xb** + **Za** + ***e***
  where **y** was the vector of cage-level records, and the distribution of the random effects was the same as for the IUAM. In this model, the cage level record, i.e., the average FS of a cage, was given to a single random animal of that cage. This was to test whether there was a benefit from fitting an animal model instead of as a sire model.

***Sire Models.*** The following three sire models were fitted:

1. Individual level bivariate sire model (**IBSiM**)
  $$\left[\begin{array}{c} y_{1}\\ y_{2} \end{array}\right]=\;\left[\begin{array}{cc} X_{1} & 0\\ 0 & X_{2} \end{array}\right]\left[\begin{array}{c} b_{1}\\ b_{2} \end{array}\right]+\left[\begin{array}{cc} Z_{1} & 0\\ 0 & Z_{2} \end{array}\right]\left[\begin{array}{c} s_{1}\\ s_{2} \end{array}\right]+\left[\begin{array}{cc} V_{1} & 0\\ 0 & V_{2} \end{array}\right]\left[\begin{array}{c} c_{1}\\ c_{2} \end{array}\right]+\left[\begin{array}{c} e_{1}\\ e_{2} \end{array}\right]$$
  Where **y**_1_ and **y**_2_ were vectors of individual records, **s**_1_ and **s**_2_ were vectors of sire effects.
  The distributional assumption of sire genetic effects was:
  $$\left[\begin{array}{c} s_{1}\\ s_{2} \end{array}\right]=N\left(\left(\begin{array}{c} 0\\ 0 \end{array}\right),\;\left[\begin{array}{cc} \sigma_{S_{1}}^{2} & \sigma_{S_{1}S_{2}}\\ \sigma_{S_{1}S_{2}} & \sigma_{S_{2}}^{2} \end{array}\right]⊗A\right)$$
  Where $\sigma_{S_{1}}^{2}$ was the variance of the sire effects for FS, $\sigma_{S_{2}}^{2}$ was the variance of the sire effects for survival, $\sigma_{S_{1}S_{2}}$ was the covariance of the sire effects between the traits, ⊗ denoted the Kronecker product of matrices, and **A** was the matrix of additive genetic relationships between individuals. The A matrix was constructed with full pedigree of the sires.
  The distribution of the cage effects and the residuals were analogous to the IBAM.
2. Cage level bivariate sire model (**CBSiM)**
  $$\left[\begin{array}{c} y_{1}\\ y_{2} \end{array}\right]=\;\left[\begin{array}{cc} X_{1} & 0\\ 0 & X_{2} \end{array}\right]\left[\begin{array}{c} b_{1}\\ b_{2} \end{array}\right]+\left[\begin{array}{cc} Z_{1} & 0\\ 0 & Z_{2} \end{array}\right]\left[\begin{array}{c} s_{1}\\ s_{2} \end{array}\right]+\left[\begin{array}{c} e_{1}\\ e_{2} \end{array}\right]$$
  Where **y**_1_ and **y**_2_ were vectors of cage level records, **s**_1_ and **s**_2_ were vectors of sire effects, and distributional assumptions for sire effects and residuals were analogous to IBSiM. Note that all cage members had the same sire.
3. Cage level univariate sire model (**CUSiM**)
  **y**_1_ = **Xb** + **Zs** + ***e***
  Where **y** was a vector of cage level records for FS, **s** was a vector of sire effects, and distributional assumptions were $s∼\;N(0,\;A\sigma_{s}^{2}),$ and $e∼\;N(0,\;I\sigma_{e}^{2})$.

### Validation

All models were validated for all four FS traits separately using a 10-fold cross-validation, where a different 10% of the cages were masked for each of the training sets. All training sets were identical across models.

Validation was based on the estimated sire effects. For each fold, estimated sire effects for FS (${\hat{s}}_{1}$) were obtained, and compared to the masked cage level phenotypes for FS (Ȳ_obs_) for that fold. The masked FS phenotypes were pre-corrected for fixed effects. Thus, the Ȳ_obs_ were the cage average residuals of a univariate cage-level model for FS that contained only the fixed effects and used the complete dataset.

***Genetic Parameters.*** Genetic parameters were estimated for each fold. As FS is affected by social-behavior and given that the records were obtained from half-sib groups with information on the sire only, the estimate of genetic variance will represent the so-called total additive variance of the trait, which includes both the direct and social genetic component (Bijma et al., 2007; Ellen et al., 2007). Thus, analogous to heritability, we calculated the ratio of total heritable variance over phenotypic variance for animal models as (Bergsma et al., 2008):

$$\hat{T^{2}}=\frac{\hat{\sigma_{A}^{2}}}{\hat{\sigma_{P}^{2}}}$$

Where $\;\hat{\sigma_{A}^{2}}$, and $\hat{\sigma_{P}^{2}}$ are the genetic and phenotypic variance estimates, respectively.

Analogous to classical heritability, for sire models we used:

$$\hat{T^{2}}=\frac{4\hat{\sigma_{S}^{2}}}{\hat{\sigma_{P}^{2}}}$$

Where $\hat{\sigma_{S}^{2}}$ is the estimated sire variance.

The phenotypic variance was calculated by the summation of $\hat{\sigma_{A}^{2}}$, the estimated residual variance ($\hat{\sigma_{e}^{2}}$), and the estimated random cage variance ($\hat{\sigma_{c}^{2}}$). For sire models, the phenotypic variance was calculated as the sum of the sire variance and the residual variance. Cage-level records include elements that are averaged over the cage members (such as the Mendelian sampling component of the breeding value and the residual) and elements that are not averaged because they are shared among cage mates, such as the genetic effect of the sire. Because of the averaged elements, $\hat{\sigma_{P}^{2}}$ of cage-level records was expected to be smaller than that of individual records.

*T^2^* was estimated for every fold. Subsequently, the estimated marginal mean (**EMMEAN**) of $\hat{T^{2}}$ for each model was calculated. EMMEAN considered the training sets across models as identical, thus, noise variance was reduced (Schrauf et al., 2021).

***Accuracy.*** Accuracy is the correlation between true breeding values and EBVs across a series of individuals (Falconer and Mackay, 1996). Instead of breeding values, here we used the sire effects (also known as transmitting abilities), which is equivalent to using sire breeding values. Because true sire effects are unknown, the masked cage-level phenotypes pre-corrected for fixed effects (Ȳ_obs_) were used as proxies of the true sire effects (Daetwyler et al., 2013). To estimate the accuracy, we divided the resulting correlation by the correlation between true sire effects and masked phenotypes (analogous to Daetwyler et al. (2013)).

$$r\left(s_{1},\;{\hat{s}}_{1}\right)=\;\frac{r\left({\hat{s}}_{1},{\bar{Y}}_{obs}\right)}{r\left(s_{1},{\bar{Y}}_{obs}\right)}$$

The $r({\hat{s}}_{1},{\bar{Y}}_{obs})$ was the EMMEAN of the correlation values for each fold, ${\bar{Y}}_{obs}\;$ is a cage-level record and ${\hat{s}}_{1}$ denotes the estimated sire effect for an FS trait. For each fold, the $r(s_{1},\;{\bar{Y}}_{obs})$ was calculated as the square root of the fraction of variance in ${\bar{Y}}_{obs}$ explained by $s_{1}$, using the estimated variance components obtained from the IBAM model for that fold, where *n* is the mean cage size.

$$r\left(s_{1},\;{\bar{Y}}_{obs}\right)=\sqrt{\frac{\frac{1}{4}\hat{\sigma_{A}^{2}}}{\frac{1}{4}\hat{\sigma_{A}^{2}}+\hat{\sigma_{c}^{2}}+\;\frac{\frac{3}{4}\hat{\sigma_{A}^{2}}+\hat{\sigma_{e}^{2}}}{n}}}$$

In the denominator, the $\frac{1}{4}\hat{\sigma_{A}^{2}}$ refers to the variance coming from the sire, which is common to the cage members and thus not divided by *n*. the $\frac{3}{4}\hat{\sigma_{A}^{2}}\;$ refers to the variance coming from the dam, and from the Mendelian sampling. The r($s_{1}$, ${\bar{Y}}_{obs}$) was calculated for each fold and then EMMEANs were obtained.

Note that, while the model used to estimate the variance components that were applied in the calculation of $r(s_{1},\;{\bar{Y}}_{obs})$ may affect the overall level of $r(s_{1},\;{\hat{s}}_{1})$, it does not impact the model comparison. This is because we used the same $r(s_{1},\;{\bar{Y}}_{obs})$ for all models in the comparison, so it is merely a fixed scalar in the equation for $r(s_{1},\;{\hat{s}}_{1})$. Hence, using the IBAM model to estimate r($s_{1}$, ${\bar{Y}}_{obs}$) does not favour the IBAM model in the model comparison.

***Expected Accuracy.*** Theoretical accuracy values of the models were calculated to obtain a comparison reference for the “observed” accuracies calculated in the previous section.

The accuracy of breeding values can be calculated as the square root of their reliability. The reliability of breeding values from single observations (or from averages) is equal to the portion of the variance in the observations explained by the genetic source of interest (e.g., Bijma and Dekkers (2022)). For example, the reliability of the EBV of a sire estimated from a progeny tests is equal to the proportion of variance in the progeny means that is explained by the sire effect. Using the variance components estimated with each of the models, we calculated expected accuracies as $\sqrt{R^{2}}$ . The formulas for Individual- and Cage-level models were as follows:

• Individual-level models
  Here the accuracy follows from the proportion of variance in the mean of the individual-level observations explained by the sire:
  ∘ IBAM and IUAM
    $$\sqrt{R^{2}}=\sqrt{\frac{\frac{1}{4}\hat{\sigma_{A}^{2}}}{\frac{1}{4}\hat{\sigma_{A}^{2}}+\frac{\frac{1}{4}\hat{\sigma_{A}^{2}}}{d}+\frac{\frac{1}{2}\hat{\sigma_{A}^{2}}}{n}+\frac{\hat{\sigma_{e}^{2}}}{n}+\frac{\hat{\sigma_{c}^{2}}}{m}}\;}$$
    Where d is the mean number of dams per sire (d=20), n is the mean number of daughters per sire (n=53) and m is the mean number of cages with daughters per sire (m=6). In the denominator, $\frac{\frac{1}{4}\hat{\sigma_{A}^{2}}}{d}$ refers to the genetic variance in the average of the breeding values of the d dams mated to the sire, $\frac{\frac{1}{2}\hat{\sigma_{A}^{2}}}{n}$ is the genetic variance in the average of the n Mendelian sampling terms, $\frac{\hat{\sigma_{c}^{2}}}{m}$ is the variance in the average of the m cage effects of the daughters of a sire, and $\frac{\hat{\sigma_{e}^{2}}}{n}$ is the variance of the average of the n residuals.
  ∘ IBSiM
    For IBSiM $\sigma_{S}^{2}$ is estimated instead of $\sigma_{A}^{2}$, $\sigma_{S}^{2}=\frac{1}{4}\sigma_{A}^{2}$ and $\sigma_{e}^{2}$ contains $\frac{3}{4}\sigma_{A}^{2}$. By substituting these relationships we find:
    $$\sqrt{R^{2}}=\sqrt{\frac{\hat{\sigma_{S}^{2}}}{\hat{\sigma_{S}^{2}}+\frac{\hat{\sigma_{S}^{2}}}{d}+\frac{2\hat{\sigma_{S}^{2}}}{n}+\frac{\hat{\sigma_{e}^{2}}-3\hat{\sigma_{S}^{2}}}{n}+\frac{\hat{\sigma_{c}^{2}}}{m}}\;}$$
• Cage-level models
  The input observation for these models were the averages per cage, so that n=m. Here we assumed that cage mates were half-sibs, ignoring the occasional full sibs that may have been present in the cage. Thus, the formulas used for cage-level models were:
  ∘ CUAM
    $$\sqrt{R^{2}}=\sqrt{\frac{\frac{1}{4}\hat{\sigma_{A}^{2}}}{\frac{1}{4}\hat{\sigma_{A}^{2}}+\frac{\frac{3}{4}\hat{\sigma_{A}^{2}}+\hat{\sigma_{e}^{2}\;}}{m}}\;}$$
    In the denominator $\frac{3}{4}\hat{\sigma_{A}^{2}}\;$ refers to the genetic variance coming from the dam and from the Mendelian sampling. $\frac{\frac{3}{4}\hat{\sigma_{A}^{2}}+\hat{\sigma_{e}^{2}\;}}{m}$ refers to the variance in the progeny average that is not due to the sire.
  ∘ CUSiM and CBSiM
    For these models, the obtained formula was:
    $$\sqrt{R^{2}}=\sqrt{\frac{\hat{\sigma_{S}^{2}}}{\hat{\sigma_{S}^{2}}+\frac{\hat{\sigma_{e}^{2}\;}}{m}}\;}$$

***Dispersion.*** In addition to the accuracy, we validated the dispersion of the estimated sire effects (sometimes also referred to as “bias”). For a BLUP estimate, the regression coefficient of a masked phenotype on the sire effect underlying that phenotype has an expected value of one. Therefore, for each fold, we regressed Ȳ_obs_ on ${\hat{s}}_{1}$. We calculated the mean regression coefficient and its standard error for every model and compared the resulting values to the expected value of one. Regression coefficients greater than one indicate under-dispersion of estimated sire effects, while regression coefficients smaller than one indicate over-dispersion.

## Results

### Genetic parameters

**Fig. 1:**
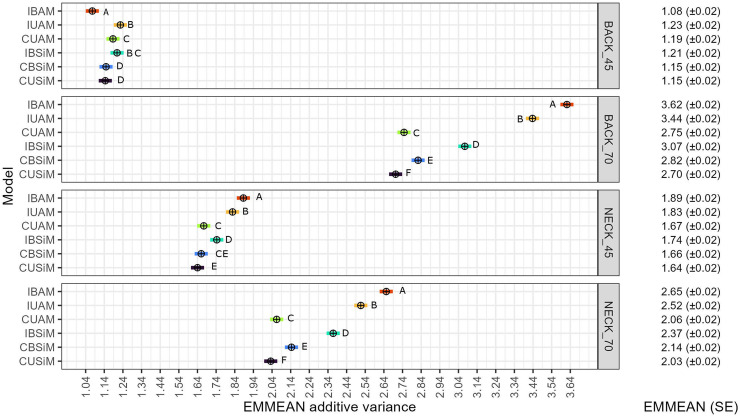
EMMEAN additive genetic variance and confidence interval estimated with each model for each trait. Within trait comparison of models’ EMMEAN additive genetic variance. EMMEANs that share the same letter are not significantly different from each other (*P* < 0.05). BACK_45: Feather damage trait from the back at 45 weeks of age; BACK_70: Feather damage trait from the back at 70 weeks of age; NECK_45: Feather damage trait from the neck at 45 weeks of age; NECK_70 Feather damage trait from the neck at 70 weeks of age. IBAM: Individual bivariate animal model; IUAM : Individual univariate animal model; CUAM : Cage univariate animal model; IBSiM: Individual bivariate sire model, CBSiM: Cage bivariate sire model; CUSiM: Cage univariate sire model. EMMEAN: estimated marginal mean of the additive variances across folds for each model. SE: Standard error of the mean.

EMMEANs of $\hat{\sigma_{A}^{2}}$ (Fig. 1) for BACK70 and NECK70 were higher than those for BACK45 and NECK45 across models. In addition, EMMEAN $\hat{\sigma_{A}^{2}}$ of individual-level models seemed slightly higher than those of cage-level models, which was most evident for BACK70 and NECK70.

Differences in $\hat{\sigma_{A}^{2}}$ were small between corresponding univariate and bivariate models (IBAM vs IUAM and CBSiM vs CUSiM) and between corresponding animal and sire models (IBAM vs IBSiM and CUAM vs CUSiM). Differences between individual-level animal models and cage-level sire models were somewhat larger, particularly for week 70 FS traits (IBAM vs CBSiM and IUAM vs CUSiM).

Within trait between models, most EMMEAN of $\hat{\sigma_{A}^{2}}$ were significantly different from each other (*P* < 0 .05). The exceptions were: CBSiM – CUSiM (BACK45), CUAM – IBSiM (BACK45), IBSiM – IUAM (BACK45), CBSiM – CUAM (NECK45), and CBSiM – CUSiM (NECK45) (Fig. 1). However, when the sire effects estimated by analogous sire and animal models (IBAM – IBSiM, and CUAM – CUSiM) were compared, a correlation of 0.99 was obtained in both cases (results not shown).

In addition, a slope of 0.49 was found in both comparisons when sire breeding values estimated with animal models were regressed on sire effects estimated with the sire models (results not shown). Note that sire models would estimate the effect that a sire has on a cage, while an animal model would estimate the complete breeding value of a sire. Hence, breeding values estimated with animal models are expected to be twice as large as the sire effects estimated with sire models, which is consistent with the slope of 0.49 that was found. Thus, no meaningful differences between EBVs from sire and animal models were found.

On average, estimated genetic standard deviations were around 1.2 for week 45 FS traits, and 1.6 for week 70 FS traits. Comparing these values to the trait averages (Table 1) shows that the trait averages are between two and three genetic standard deviations away from the minimum trait value (10, representing a perfect feather score). This indicates that genetic variation is substantial compared to the trait level. In other words, three genetic standard deviations of improvement would fully remove feather damage, apart from non-genetic variation.

However, the estimation of variance components for survival was not robust with IBAM. The estimation of the residual covariance between FS and survival had to be set to zero, and the $\hat{\sigma_{A}^{2}}$ for survival was very close to zero (data not shown) and the estimated genetic correlation between FS and survival was zero (Table S1 and S2). On the other hand, the residual covariance was easily estimated when using IBSiM and CBSiM. The $\hat{\sigma_{A}^{2}}$ for survival with these two models was larger than that estimated with IBAM, and the genetic correlations estimates between FS and survival ranged from ∼0.2 to ∼0.6 (Table S1 and S2).

**Fig. 2:**
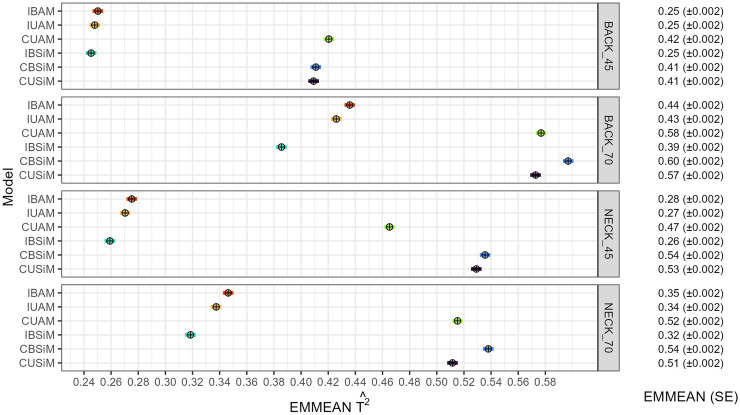
EMMEAN $\hat{T^{2}}$ and confidence interval estimated with each model for each trait. BACK_45: Feather damage trait from the back at 45 weeks of age; BACK_70: Feather damage trait from the back at 70 weeks of age; NECK_45: Feather damage trait from the neck at 45 weeks of age; NECK_70 Feather damage trait from the neck at 70 weeks of age. IBAM: Individual bivariate animal model; IUAM: Individual univariate animal model; CUAM: Cage univariate animal model; IBSiM: Individual bivariate sire model; CBSiM: Cage bivariate sire model; CUSiM: Cage univariate sire model. EMMEAN: estimated marginal mean of the $\hat{T^{2}}$ across folds for each model. SE: Standard error of the mean.

Fig. 2 shows the estimated EMMEANs of $T^{2}$. In all but one case, the $\hat{T^{2}}$ estimated for BACK70 and NECK70 were higher than those for BACK45 and NECK45. Also, the $\hat{T^{2}}$ from cage-level models were consistently larger than those of individual records models. The latter was related to a smaller $\hat{\sigma_{P}^{2}}$ for cage-level models. Table S1 shows the EMMEANs of all variance components of all models and traits. The $\hat{\sigma_{P}^{2}}$ of individual records models were approximately twice as large than those of cage-level models, which more than compensated for the smaller $\hat{\sigma_{A}^{2}}$ of cage-level models, and thus resulted in larger $\hat{T^{2}}$.

### Accuracy

**Fig. 3:**
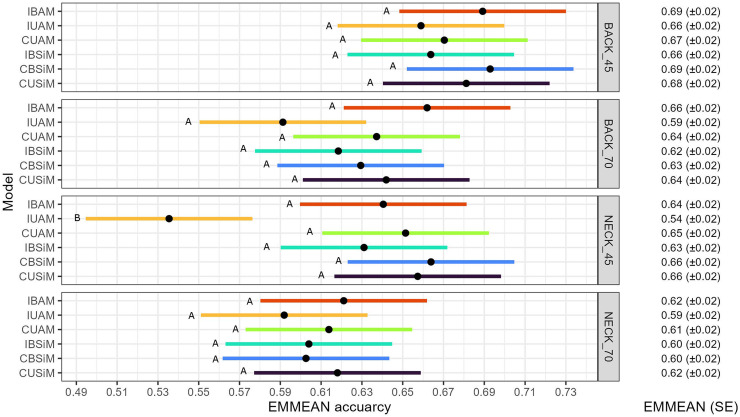
EMMEAN accuracy and confidence interval from each model for each trait. Within trait comparison of the models’ EMMEAN accuracy, EMMEANS with different letters are significantly different from each other (*P* < 0.05). BACK_45: Feather damage trait from the back at 45 weeks of age; BACK_70: Feather damage trait from the back at 70 weeks of age; NECK_45: Feather damage trait from the neck at 45 weeks of age; NECK_70 Feather damage trait from the neck at 70 weeks of age. IBAM: Individual bivariate animal model; IUAM: Individual univariate animal model; CUAM: Cage univariate animal model; IBSiM: Individual bivariate sire model; CBSiM: Cage bivariate sire model; CUSiM: Cage univariate sire model. EMMEAN: estimated marginal mean of the accuracy obtained across folds for each model. SE: Standard error of the mean.

Fig. 3 shows EMMEANs of accuracies from the cross-validation for all models for the four traits. The overall level of accuracy of ∼0.6 indicates that the EBVs correlate moderately well with the true breeding values (Oldenbroek and Waaij, 2014), suggesting that the selection of these EBV should result in genetic improvement of FS. A comparison of accuracies within trait showed that values were always lowest for the IUAM. Within trait NECK45 the accuracy for the IUAM was the only that was significantly different from others (*P* < 0.05). Whitin the other traits no significant differences were found when the accuracies of the models were compared. Apart from the IUAM, no clear superiority of bivariate models over univariate models was found, nor of animal models over sire models.

The EMMEAN accuracy of each model for BACK45 was compared to that for BACK70, and the same was done for NECK45 and NECK70. With an alpha of 0.05, no significant difference was found. Thus, the accuracy of EBVs did not depend significantly on the moment of the scoring.

### Dispersion

**Fig. 4:**
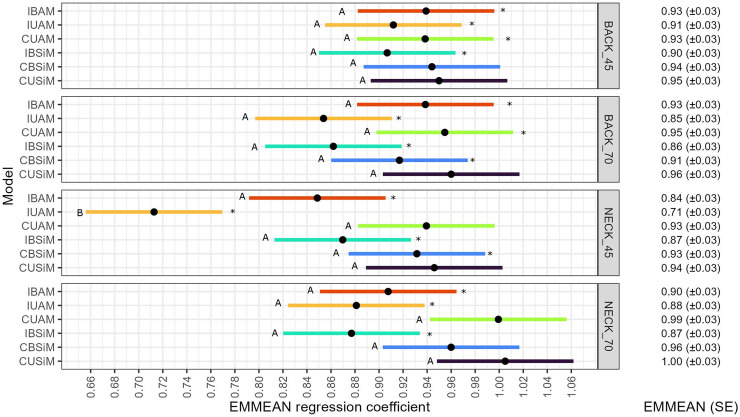
EMMEAN regression coefficient and confidence interval from each model for each trait Within trait comparison of the models’ EMMEAN regression coefficient. EMMEANS with different letters are significantly different from each other (*P* < 0.05). EMMEANS marked with “ * ” are significantly different from one (*P* < 0.05). BACK_45: Feather damage trait from the back at 45 weeks of age; BACK_70: Feather damage trait from the back at 70 weeks of age; NECK_45: Feather damage trait from the neck at 45 weeks of age; NECK_70 Feather damage trait from the neck at 70 weeks of age. IBAM: Individual bivariate animal model; IUAM: Individual univariate animal model; CUAM: Cage univariate animal model; IBSiM: Individual bivariate sire model; CBSiM: Cage bivariate sire model; CUSiM: Cage univariate sire model. EMMEAN: estimated marginal mean of the regression coefficient obtained across folds for each model. SE: Standard error of the mean.

The majority of EMMEAN regression coefficients were significantly smaller than one (*P* < 0.05, Fig. 4). Thus, slight to moderate over-dispersion of estimated genetic effects was observed for the majority of the models. Only for CUSiM, the regression coefficient was not significantly different from one for all traits. Cage-level models showed a tendency to have a smaller deviation from one than individual-level models.

A similar pattern in the results was found for EMMEAN regression coefficients (Fig. 4) as for EMMEAN accuracies (Fig. 3). In a within-trait comparison, only the EMMEAN regression coefficient of IUAM for NECK45 was significantly smaller than the others (*P* < 0.05). Hence, the joint results for accuracy and dispersion show that IUAM performed worst, and indicate that a bivariate analysis of FS and survival is beneficial for the individual-level models. However, no such benefit was observed for the cage-level models.

## Discussion

In this study, we proposed and compared six models for the genetic analysis of FS in recurrent test data of laying hens. We fitted three animal models and three sire models, both with univariate analysis of FS and bivariate analysis of FS and survival to account for possible bias due to pre-selection for the latter. The performance of each model was assessed by comparing the marginal means of the accuracy and dispersion obtained in a 10-fold cross-validation.

Overall, there was no evident superiority of either animal or sire models. This is probably because the dams of individuals were not known, thus, no extra information was given when fitting an animal model. Additionally, the lack of evident differences between bivariate and univariate analysis (except for IUAM) could be related to low mortality and FS not varying much over time (Table 1).

The issues when estimating covariances between FS and survival with the IBAM were attributed to the lack of variability in survival records. All individuals that had a score for FS had the same “2″ as the survival record, which resulted in very little information to estimate the residual covariance between FS and survival. This may explain why FS-survival residual covariance had to be set to a fixed value. Conversely, with IBSiM, ¾ of the $\sigma_{A}^{2}$ were considered as part of the residual variance, which potentially gave enough information for the model to estimate the residual covariance.

In our analysis, we did not explicitly model social genetic effects. In general, accounting for the receiver and performer effect is important when analyzing traits affected by behavioral interactions among individuals, because “social environment” includes part of the heritable variance in the trait. When the group members are not equally related, it is possible to estimate social genetic effects (IGEs) and direct genetic effects (DGEs) from individual trait observations and pedigree information (Ellen et al., 2008). Such models, are for example, used to estimate DGE and IGE for tail damage in pigs (Canario and Flatres-Grall, 2018). However, in our data, individuals were kept in groups sired by the same father and individuals had unknown dams. Thus, the pedigree information of all cage members was identical, and therefore DGEs and IGEs are indiscernible. Nonetheless, a classical linear mixed model applied to our data will produce estimates of total breeding values and total genetic variance (Peeters et al., 2013; Brinker et al., 2017). The “total breeding value” includes the direct genetic effect of an individual on its own phenotype, plus its indirect genetic effect on the phenotypes of its partners (Bijma, 2010). In our case, as cage members were half-sibs, our analysis captured both performer and receiver effects on FS. Hence, this could explain the relatively high values of our $T^{2}$ estimates compared to common values for the ordinary direct $h^{2}$ for behavior-related discrete phenotypes.

As mentioned before, there was a big difference between $\hat{\sigma_{P}^{2}}\;$from individual record models and cage-level models (Table S1 and S2). The $\hat{\sigma_{P}^{2}}$ of an average is smaller as the summation of variance components is divided over the number of individuals in a group (Biscarini et al., 2010; Bennett et al., 2014). In our case, as individuals were related, some components of the phenotype were shared among cage mates, while others were not. For example, as all individuals had a different dam and Mendelian sampling term, the genetic effects of the dams should be averaged over *n*, thus contributing $\frac{\frac{1}{4}\hat{\sigma_{A}^{2}}}{n}\;$to $\hat{\sigma_{P}^{2}}$ . The same applied to $\hat{\sigma_{e}^{2}}$ and the $\frac{1}{2}\hat{\sigma_{A}^{2}}$ from Mendelian sampling. Hence, $\hat{\sigma_{P}^{2}}$ was smaller for cage-level models. Since $T^{2}$ is the ratio of the total heritable variance over the phenotypic variance (Bergsma et al., 2008), its value is affected by averaging (i.e. the dams effect, the Mendlian sampling and the residual) in the calculation of the denominator. Hence, this resulted in larger $T^{2}$ values for cage-level models (Fig. 2). However, the use of averages did not improve the accuracy of the breeding value estimation, as there was no significant difference among models, except with IUAM for NECK45 (Fig. 3).

As the denominator if $T^{2}$ is affected by averaging, it is more appropriate to compare $\hat{\sigma_{A}^{2}}$. We do not understand why the $\hat{\sigma_{A}^{2}}$ of individual-level models were slightly higher than that of cage-level models. For both models, the information to estimate the genetic variance comes from between-cage covariances. This is obvious for cage-level models as they have only a single record per cage, while it also applies to individual-level models because within-cage covariances are fully confounded with the random cage effect. Because both models estimate the genetic variance from between-cage covariances (mainly between cages containing half-sibs), we would have expected similar estimates of $\hat{\sigma_{A}^{2}}$. Maybe the difference originates from variations in cage size or from deviations from normality, which are more serious for the individual-level records, but we are not sure.

The large $\hat{\sigma_{A}^{2}}$ and $T^{2}$ obtained for BACK70 and NECK70 (Fig. 1, Fig. 2) compared to BACK45 and NECK45 can probably be attributed to an underlying continuous liability for FS while phenotypic records were discrete, together with a difference in mean trait value between weeks 45 and 70. The liability is an invisible underlying continuous scale for a binary, or discrete variable, and is typically considered to be normally distributed (Wright, 1934; Falconer and Mackay, 1996). The heritability of such variables can be defined on the liability scale, and on the observed scale (Dempster and Lerner, 1950, Robertson, 1950). For binary traits, the observed scale heritability is lower when the mean trait value is closer to zero or one, and higher for intermediate trait values (Dempster and Lerner, 1950, Robertson, 1950). This same phenomenon will also occur for discrete variables with more than two classes, albeit to a lesser extent (Thompson et al., 1985). In this study, the mean FS for BACK45 and NECK45 were closer to the lower limit of the scale (10), while for BACK70 and NECK70, FS values were more intermediate (Table 1). We expect this mechanism largely explains the observed difference in estimated magnitudes of $\hat{\sigma_{A}^{2}}$ and $T^{2}$ between 45 and 70 weeks of age.

Accuracies of EBVs are of high importance. More accurate estimates translate into greater genetic gains and also more reliable predictions of genetic gains. Therefore, we compared our accuracies to theoretically expected accuracies based on the estimated genetic parameters. For example, for trait BACK45 and CBSiM, an expected accuracy of 0.64 was found (Table S3), in the 10-fold validation, the accuracy obtained for CBSiM BACK45 was 0.69 (Fig. 3), which agrees quite well with the theoretically expected value of 0.64.

Because we used the estimated genetic parameters to calculate expected accuracies, these were affected by the differences in the $\hat{\sigma_{A}^{2}}$. This could explain the relatively small differences between obtained and expected accuracy that was observed for cage level models, and mainly for trait BACK70 (Table S3), for which higher $\hat{\sigma_{A}^{2}}$ was estimated with the models (Fig. 1).

There was no evident reason for IUAM's accuracy for NECK45 to be significantly lower than the rest (Fig. 3). All validation datasets were identical across models, and the correlation between “real” breeding values and pre-corrected cage-level phenotypes was standardized across models because all EBVs were compared to the same pre-corrected phenotypes. Therefore, the difference in accuracy was not due to the differences in the heritable part of the phenotype explained by the models (i.e., $r(s_{1},\;{\bar{Y}}_{obs})$) (Daetwyler et al., 2013; Schrauf et al., 2021) or given by differences in which information was masked during the validation. In any case, the estimates obtained with IUAM for NECK45 also had the largest overdispersion among models (Fig. 4).

Next to accuracy, a correct dispersion of EBVs is important for response to selection. A regression coefficient that differs from one means that there is an over or under-dispersion of EBVs. When selecting univariately, incorrect dispersion does not alter the ranking of EBVs, it only alters their scale. However, in a multi-trait selection index, incorrect dispersion can reduce accuracy because the correlation between aggregate genotype and index is reduced (Sales and Hill, 1976; Vandepitte et al., 1977). In our analysis, models with cage-level records showed a tendency for less over-dispersion.

## Conclusions

• Feather score showed a large total heritable variation and EBVs had moderate accuracy, together indicating good prospects for genetic improvement based on recurrent test data.
• The Individual Univariate Animal Model (IUAM) showed the poorest performance, both for accuracy and dispersion.
• Apart from the IUAM, no clear superiority regarding accuracy was found between bivariate and univariate models, nor between animal models and sire models.
• Cage-level models showed less over-dispersion.
• The dispersion for CUSiM was not significantly different from one for all traits.

Considering these results, from the tested models, CUSiM (cage level univariate sire model) was considered the best model for the analysis of feather score using information from recurrent testing facilities.

## Disclosures

The authors declare the following financial interests/personal relationships which may be considered as potential competing interests:

Tzayhri Osorio Gallardo reports financial support was provided by Dutch Research Council (NWO-TTW). Tzayhri Osorio Gallardo reports equipment, drugs, or supplies was provided by Hendrix Genetics BV. If there are other authors, they declare that they have no known competing financial interests or personal relationships that could have appeared to influence the work reported in this paper.

## Appendix Supplementary materials

Image, application 1
