# Supplementary material for: Comparison of linear mixed models for genetic feather score analysis in laying hens kept in recurrent testing facilities
Source: Poult Sci. 2025 Jan 20;104(2):104833. doi: 10.1016/j.psj.2025.104833 (PMC11810823; doi:10.1016/j.psj.2025.104833)
Supplement: Supplementary file 1 [file mmc1.docx]

## Supplementary material

Table S1. EMMEANs of variance components of feather score and feather score-survival genetic correlation from all models for BACK traits

|  | EMMEAN variance components of FS and FS-survival genetic correlation for BACK traits | | | | | | | | | |  |
| --- | --- | --- | --- | --- | --- | --- | --- | --- | --- | --- | --- |
| MODELS | 45 weeks | | | | | | 70 weeks | | | | |
|  | $\hat{\sigma_{A}^{2}}$ or $\hat{\sigma_{S}^{2}}$ | $\hat{\sigma_{e}^{2}}$ | $\hat{\sigma_{c}^{2}}$ | $\hat{\sigma_{P}^{2}}$ | ρ(FS, Surv) |  | $\hat{\sigma_{A}^{2}}$ or $\hat{\sigma_{S}^{2}}$ | $\hat{\sigma_{e}^{2}}$ | $\hat{\sigma_{c}^{2}}$ | $\hat{\sigma_{P}^{2}}$ | ρ(FS, Surv) |
| IBAM | 1.30  (±0.01) | 1.76  (±0.01) | 1.97  (±0.001) | 5.04  (±0.003) | 0  (±0.02) |  | 3.62  (±0.01) | 1.78  (±0.01) | 2.91  (±0.001) | 8.31  (±0.003) | 0  (±0.02) |
| IUAM | 1.23  (±0.01) | 1.74  (±0.01) | 1.98  (±0.001) | 4.95  (±0.003) | - |  | 3.44  (±0.01) | 1.64  (±0.01) | 2.99  (±0.001) | 8.07  (±0.003) | - |
| CUAM | 1.19  (±0.01) | 1.63  (±0.01) | 0 | 2.82  (±0.003) | - |  | 2.75  (±0.01) | 2.01  (±0.01) | 0 | 4.76  (±0.003) | - |
| IBSiM | 0.30  (±0.01) | 2.64  (±0.01) | 1.98  (±0.001) | 4.93  (±0.003) | -0.60  (±0.02) |  | 0.77  (±0.01) | 4.16  (±0.01) | 3.04  (±0.001) | 7.97  (±0.003) | -0.33  (±0.02) |
| CBSiM | 0.29  (±0.01) | 2.51  (±0.01) | 0 | 2.80  (±0.003) | -0.62  (±0.02) |  | 0.71  (±0.01) | 4.02  (±0.01) | 0 | 4.73  (±0.003) | -0.33  (±0.02) |
| CUSiM | 0.29  (±0.01) | 2.51  (±0.01) | 0 | 2.80  (±0.003) | - |  | 0.68  (±0.01) | 4.04  (±0.01) | 0 | 4.72  (±0.003) | - |

FS: Feather Score; Surv: Survival; IBAM: Individual bivariate animal model; IUAM: Individual univariate animal model; CUAM: Cage univariate animal model; IBSiM: Individual bivariate sire model; CBSiM: Cage bivariate sire model; CUSiM: Cage univariate sire model. $\sigma_{A}^{2}$: estimated genetic variance; $\hat{\sigma_{S}^{2}}$: estimated genetic variance of the sire; $\hat{\sigma_{e}^{2}}$: estimated residual variance; $\hat{\sigma_{c}^{2}}$: estimated random cage variance; $\hat{\sigma_{P}^{2}}$: estimated phenotypic variance. SE: Standard error of the mean.

Table S2. EMMEANs of variance components of feather score and feather score-survival genetic correlation from all models for NECK traits

| EMMEAN variance components of FS and FS-survival genetic correlation for NECK traits | | | | | | | | | | | | |
| --- | --- | --- | --- | --- | --- | --- | --- | --- | --- | --- | --- | --- |
| MODELS | 45 Weeks | | | | | | | 70 Weeks | | | | |
|  |  | $\hat{\sigma_{A}^{2}}$ or $\hat{\sigma_{S}^{2}}$ | $\hat{\sigma_{e}^{2}}$ | $\hat{\sigma_{c}^{2}}$ | $\hat{\sigma_{P}^{2}}$ | ρ(FS, Surv) |  | $\hat{\sigma_{A}^{2}}$ or $\hat{\sigma_{S}^{2}}$ | $\hat{\sigma_{e}^{2}}$ | $\hat{\sigma_{c}^{2}}$ | $\hat{\sigma_{P}^{2}}$ | ρ(FS, Surv) |
| IBAM |  | 1.89 (±0.01) | 3.07 (±0.01) | 1.90 (±0.001) | 6.85 (±0.003) | 0  (±0.02) |  | 2.65 (±0.01) | 2.88 (±0.01) | 2.13 (±0.001) | 7.66 (±0.003) | 0  (±0.02) |
| IUAM |  | 1.83 (±0.01) | 3.00 (±0.01) | 1.93 (±0.001) | 6.76 (±0.003) | - |  | 2.52 (±0.01) | 2.72 (±0.01) | 2.22 (±0.001) | 7.46 (±0.003) | - |
| CUAM |  | 1.67 (±0.01) | 1.46 (±0.01) | 0 | 3.13 (±0.003) | - |  | 2.06 (±0.01) | 1.94 (±0.01) | 0 | 4.01 (±0.003) | - |
| IBSiM |  | 0.44 (±0.01) | 4.36 (±0.01) | 1.94 (±0.001) | 6.73 (±0.003) | -0.55  (±0.02) |  | 0.59 (±0.01) | 4.57 (±0.01) | 2.28 (±0.001) | 7.43 (±0.003) | -0.20  (±0.02) |
| CBSiM |  | 0.41 (±0.01) | 2.68 (±0.01) | 0 | 3.10 (±0.003) | -0.55  (±0.02) |  | 0.54 (±0.01) | 3.45 (±0.01) | 0 | 3.99 (±0.003) | -0.18  (±0.02) |
| CUSiM |  | 0.41 (±0.01) | 2.69 (±0.01) | 0 | 3.10 (±0.003) | - |  | 0.51 (±0.01) | 3.46 (±0.01) | 0 | 3.97 (±0.003) | - |

FS: Feather Score; Surv: Survival; IBAM: Individual bivariate animal model; IUAM: Individual univariate animal model; CUAM: Cage univariate animal model; IBSiM: Individual bivariate sire model; CBSiM: Cage bivariate sire model; CUSiM: Cage univariate sire model. $\sigma_{A}^{2}$: estimated genetic variance; $\hat{\sigma_{S}^{2}}$: estimated genetic variance of the sire; $\hat{\sigma_{e}^{2}}$: estimated residual variance; $\hat{\sigma_{c}^{2}}$: estimated random cage variance; $\hat{\sigma_{P}^{2}}$: estimated phenotypic variance. SE: Standard error of the mean.

Table S3. Comparison of expected and obtained accuracy from all models for all traits

|  | Obtained and expected accuracies (SE) | | | | | | | | |
| --- | --- | --- | --- | --- | --- | --- | --- | --- | --- |
|  | BACK | | | | NECK | | | | |
|  | 45 Weeks | | 70 Weeks | | 45 Weeks | | | 70 Weeks | |
| Model | **Obtained accuracy** | **Expected accuracy** | **Obtained accuracy** | **Expected accuracy** | **Obtained accuracy** | **Expected accuracy** | **Obtained accuracy** | | **Expected accuracy** |
| IBAM | 0.69  (±0.02) | 0.67 | 0.66  (±0.02) | 0.78 | 0.64  (±0.02) | 0.73 | 0.62  (±0.02) | | 0.77 |
| IUAM | 0.66  (±0.02) | 0.66 | 0.59  (±0.02) | 0.77 | 0.53  (±0.02) | 0.73 | 0.59  (±0.02) | | 0.75 |
| CUAM | 0.67  (±0.02) | 0.64 | 0.64  (±0.02) | 0.76 | 0.65  (±0.02) | 0.73 | 0.61  (±0.02) | | 0.71 |
| IBSiM | 0.66  (±0.02) | 0.67 | 0.62  (±0.02) | 0.74 | 0.63  (±0.02) | 0.71 | 0.60  (±0.02) | | 0.74 |
| CBSiM | 0.69  (±0.02) | 0.64 | 0.63  (±0.02) | 0.72 | 0.66  (±0.02) | 0.69 | 0.60  (±0.02) | | 0.69 |
| CUSiM | 0.68  (±0.02) | 0.64 | 0.64  (±0.02) | 0.71 | 0.66  (±0.02) | 0.69 | 0.62  (±0.02) | | 0.68 |

IBAM: Individual bivariate animal model; IUAM: Individual univariate animal model; CUAM: Cage univariate animal model; IBSiM: Individual bivariate sire model; CBSiM: Cage bivariate sire model; CUSiM: Cage univariate sire model. ^1^ The values closest to one are in bold. SE: Standard error of the mean.
